# Hyperactivity of Hypothalamic-Pituitary-Adrenal Axis Due to Dysfunction of the Hypothalamic Glucocorticoid Receptor in Sigma-1 Receptor Knockout Mice

**DOI:** 10.3389/fnmol.2017.00287

**Published:** 2017-09-06

**Authors:** Tingting Di, Suyun Zhang, Juan Hong, Tingting Zhang, Ling Chen

**Affiliations:** ^1^State Key Laboratory of Reproductive Medicine, Nanjing Medical University Nanjing, China; ^2^Department of Physiology, Nanjing Medical University Nanjing, China

**Keywords:** sigma-1 receptor (σ_1_R), hypothalamic-pituitary-adrenal (HPA) axis, glucocorticoid receptor (GR), protein kinase C (PKC), depression

## Abstract

Sigma-1 receptor knockout (σ_1_R-KO) mice exhibit a depressive-like phenotype. Because σ_1_R is highly expressed in the neuronal cells of hypothalamic paraventricular nuclei (PVN), this study investigated the influence of σ_1_R deficiency on the regulation of the hypothalamic-pituitary-adrenocortical (HPA) axis. Here, we show that the levels of basal serum corticosterone (CORT), adrenocorticotropic hormone (ACTH) and corticotrophin releasing factor (CRF) as well as the level of *CRF* mRNA in PVN did not significantly differ between adult male σ_1_R-KO mice and wild-type (WT) mice. Acute mild restraint stress (AMRS) induced a higher and more sustainable increase in activity of HPA axis and CRF expression in σ_1_R-KO mice. Percentage of dexamethasone (Dex)-induced reduction in level of CORT was markedly attenuated in σ_1_R^−/−^ mice. The levels of glucocorticoid receptor (GR) and protein kinase C (PKC) phosphorylation were reduced in the PVN of σ_1_R-KO mice and σ_1_R antagonist NE100-treated WT mice. The exposure to AMRS in σ_1_R-KO mice induced a stronger phosphorylation of cAMP-response element binding protein (CREB) in PVN than that in WT mice. Intracerebroventricular (i.c.v.) injection of PKC activator PMA for 3 days in σ_1_R-KO mice not only recovered the GR phosphorylation and the percentage of Dex-reduced CORT but also corrected the AMRS-induced hyperactivity of HPA axis and enhancement of *CRF* mRNA and CREB phosphorylation. Furthermore, the injection (i.c.v.) of PMA in σ_1_R-KO mice corrected the prolongation of immobility time in forced swim test (FST) and tail suspension test (TST). These results indicate that σ_1_R deficiency causes down-regulation of GR by reducing PKC phosphorylation, which attenuates GR-mediated feedback inhibition of HPA axis and facilitates the stress response of HPA axis leading to the production of depressive-like behaviors.

## Introduction

Sigma-1 receptors (σ_1_R) are highly expressed in regions of the brain that are involved in emotion and neuropsychiatric disorders (Hayashi and Su, 2004), and σ_1_R agonists are a class of drugs for the treatment of depression (Urani et al., 2001) and anxiety (Longone et al., 2011). Specifically, preclinical studies have shown that targeting σ_1_R alone is sufficient to produce antidepressant-like effects, and σ_1_R agonists exhibited a stronger antidepressant effect in clinical trials in humans (Ishikawa et al., 2007). Moreover, σ_1_R knockout (σ_1_R-KO) mice exhibit depressive-like behaviors (Chevallier et al., 2011).

Several lines of evidence suggest that the hypothalamic-pituitary-adrenocortical (HPA) axis is often hyperactive in patients suffering from major depression, and this axis has been implicated the pathophysiology of this disease (Barden, 2004). Moreover, circulating glucocorticoids are critical to recovery from stress conditions because they can inhibit the production of corticotrophin releasing hormone (CRF) and the pituitary release of adrenocorticotropic hormone (ACTH; Arnett et al., 2011). The synthetic glucocorticoids and dexamethasone (Dex) are less potent in patients with depression than in healthy subjects (Heuser et al., 1996), thus impairing feedback inhibition mechanism is well known to induce HPA axis hyperactivity in major depression (Holsboer, 2000). Partially knocking out glucocorticoid receptor (GR) gene expression in mice decreases the GR-mediated feedback inhibition of the HPA axis. GR deletion can increase stress-induced HPA axis activation (Vincent and Jacobson, 2014). As demonstrated previously, antidepressant drugs can enhance the GR-mediated inhibition of HPA axis by increasing the expression of the GR, which decreases cortisol/corticosterone (CORT) levels (Budziszewska, 2002). Immunohistochemistry experiments showed high level of σ_1_R in the hypothalamus (Phan et al., 2003), and high hybridization signals of σ_1_R are observed in hypothalamic paraventricular nuclei (PVN; Kitaichi et al., 2000). However, the effects of σ_1_R on the regulation of the HPA axis have not yet been reported.

Intracellular σ_1_R activation induces the translocation of this receptor from the endoplasmic reticulum to the plasma membrane, where it regulates membrane-bound signal transduction, including the activation of protein kinase C (PKC) β1 and β2 isoforms (Morin-Surun et al., 1999). The activation of σ_1_R can induce PKC phosphorylation (Abou-Lovergne et al., 2011). GR function is modulated by phosphorylation, and binding glucocorticoids or Dex can induce the phosphorylation of GR (Brossaud et al., 2017). Specifically, PKC directly or indirectly phosphorylates the membrane-associated GR (Ser-234; Kotitschke et al., 2009), and a nongenomic GR-mediated PKC pathway has been associated with the glucocorticoid-induced rapid inhibition of ACTH secretion (John et al., 2002). GRs are ligand-dependent transcription factors that bind to a specific DNA sequence (glucocorticoid-responsive element-GRE) and regulate the expression of many target genes (Budziszewska, 2002). Although the CRF promoter does not contain a classical GRE consensus site, it contains a specific inhibitory region of CRF promoter activity by glucocorticoids (Malkoski and Dorin, 1999). Moreover, the glucocorticoid-induced suppression of CRF promoter activity is mediated by binding between bp 2248 and 2233 of the CRF promoter in hypothalamic cells (Kageyama et al., 2011). The GR plays a key role in the glucocorticoid-induced inhibition of CRF gene transcription (Morin-Surun et al., 1999). The activation of σ_1_R may increase PKA activity via the PKC signaling pathway (Fu et al., 2010). Inhibition of PKA can block forskolin-induced CRF promoter activity in hypothalamic cells (Agarwal et al., 2005). The activation of PKA leads to the binding of cAMP-response element binding protein (CREB) to the CRE on the CRF promoter (Kageyama and Suda, 2010). Stress induces rapid CREB phosphorylation, which enhances the interaction of phosphorylated CREB with CRE of the CRF gene promoter. Glucocorticoids can suppress CREB phosphorylation resulting in the feedback inhibition of CRF-biosynthesis (van der Laan et al., 2008). Therefore, it is of great interest to investigate whether the σ_1_R deficiency in CRF neurons via down-regulation of PKC reduces the GR activity, which affects the GR-mediated feedback inhibition of the HPA axis and stress-induced CRF biosynthesis.

In the present study, we used adult male σ_1_R-KO mice and investigated the influence of σ_1_R deficiency on the activity of the HPA axis and CRF biosynthesis under basal conditions or after of exposure to a 15 min acute mild restraint stress (AMRS). We further examined the phosphorylation and expression of GR and explored the involvement of PKC and PKA-CREB signaling pathways in the GR-mediated feedback inhibition of the HPA axis and stress-induced CRF biosynthesis. Finally, we analyzed the causal link between the activity of HPA axis and the depressive-like phenotype in σ_1_R-KO mice. Our results indicate that σ_1_R deficiency reduces the GR-mediated feedback inhibition of the HPA axis and facilitates the stress response of the HPA axis *via* the down-regulation of PKC signaling, which results in hyperactivity of HPA axis to induce the production of depressive-like phenotype.

## Materials and Methods

### Mice

This study was carried out in accordance with the recommendations of experimental animal guidelines, Laboratory Animal Research Institute. The protocol was approved by the Institutional Animal Care and Ethical Committee of the Nanjing Medical University. All efforts were made to minimize animal suffering and to reduce the number of animals used. The σ_1_R KO (σ_1_R^−/−^) mice were generated and characterized as described previously (Sabino et al., 2009). Heterozygote Oprs1 mutant (+/−) Oprs1^Gt(IRESBetageo)33Lex^ embryos on a C57BL/6J × 129S/SvEv mixed background were obtained from the Mutant Mouse Resource Regional Center (MMRRC) and implanted into females C57BL/6J mice (Jackson Laboratories, Bar Harbor, ME, USA) at The Scripps Research Institute. Twelve-week-old male null mutant mice (Oprs1−/−, σ_1_R^−/−^ mice) and their age-matched wild-type (+/+, WT) littermates were employed at the beginning of the experiment. Animals were housed in plastic cages at 23 ± 2°C and 55% relative humidity with a 12:12 h light/dark cycle starting at 07:00 h in the Animal Research Center of Nanjing Medical University. The mice were group-housed (3–4/cage) together with same genotypes (Sabino et al., 2009). Water and food were given *ad libitum*.

### Administration of Drugs

PKA inhibitor H89 and PKC inhibitor GF109203X were purchased from Sigma-Aldrich (St. Louis, MO, USA), and PKC activator PMA was obtained from Medchemexpress. All the drugs were dissolved in dimethyl sulfoxide (DMSO) and diluted in 0.9% sterile saline to a final concentration of 0.5% DMSO NE-100, a σ_1_R antagonist, was kindly supplied by Taisho Pharmaceutical Co. (Ltd, Tokyo, Japan) and dissolved in 0.9% sterile saline. NE100 (0.15 nmol/mouse; Yang et al., 2011), H89 (20 nmol/mouse; Zeni et al., 2012), GF109203X (50 ng/mouse) or PMA (480 pmol/mouse; Kim et al., 2000) was injected into the right ventricle in a volume of 3 μl/day. For repeated intracerebroventricular (i.c.v.) injections of drugs, the mice were anesthetized with chloral hydrate (400 mg/kg, i.p.) and then placed into a stereotaxic instrument (Stoelting, Wood Dale, IL, USA). A small hole (2 mm diameter) was drilled in the skull using a dental drill. A guide cannula (26-gauge, Plastics One, Roanoke, VA, USA) was implanted into the right lateral ventricle (0.3 mm posterior, 1.0 mm lateral, and 2.5 mm ventral to bregma) and anchored to the skull with 3 stainless steel screws and dental cement (Wang et al., 2015). On day 3 after the surgery, the dummy cannula was removed from the guide cannula and then replaced with infusion cannulas (30 gauge) connected to a stepper-motorized micro-syringe (Stoelting, Wood Dale, IL, USA) by polyethylene tubing (PE10; Becton Dickinson, Sparks, MD, USA). Mice infused with an equal volume of vehicle (0.5% DMSO) served as the control group. After 2% Evans Blue (0.5 μl) was injected, the mice were sacrificed by an overdose of chloral hydrate, and coronal sections (400 μm) were cut using a cryostat to validate the injection site.

### Behavioral Examination

Three different behavioral tests were carried out (09:00–14:00 h) under following sequence: open-field test (OFT) → forced swim test (FST) → tail suspension test (TST). The order of testing was chosen such that test involving low stress level (OFT) preceded those involving medium stress level (FST) and high stress level (TST; Zhou et al., 2014). The OFT and the FST was spaced by at least 24 h, while the FST and the TST was spaced by at least 48 h, because the elevated basal morning plasma CORT levels return to baseline as early as 48 h after the start of stressor exposure (Reber et al., 2007). Spontaneous activity was examined using an OFT. The behavioral measure in FST or TST was the duration of immobility, interpreted as behavioral despair (Zhang S. et al., 2017). These behavioral tests were recorded by a video monitor (Winfast PVR; Leadtek Research Inc., Fremont, CA, USA). The behavioral results were analyzed using TopScan Lite 2.0 (Clever Sys, Reston, VA, USA).

#### Open-Field Test (OFT)

Each mouse was placed in a clear, open-top, square Plexiglas box (60 cm × 60 cm × 40 cm) with 15 lux lighting and allowed to freely explore for 5 min. Traveled distance were measured within 5 min (Dere et al., 2004).

#### Forced Swim Test (FST)

The FST was performed as described previously (Zhang B. et al., 2017). Briefly, swim sessions were conducted by placing mice in plastic cylinders (diameter 12 cm, height 24 cm) filled with water (23–25°C) to a height of 20 cm. The mouse was considered immobile when it stopped struggling and moved only to remain floating in the water, keeping its head above the water.

#### Tail Suspension Test (TST)

Mice were suspended by the tail using adhesive tape to a rod 60 cm above the floor as described previously (Zhou et al., 2014). The total duration of immobility during a 6 min test was scored.

### Assessment of Serum Hormones

To examine the basal activity of HPA axis, the morning basal levels of CORT, ACTH and CRF were measured (Reber et al., 2007). The blood samples were taken at 08:00–10:00 h (Uschold-Schmidt et al., 2013). Serum (total 300 μl per mouse) was separated by centrifugation at 4°C (Angle Rotor, Thermo Scientific, USA) and stored at −80°C until the assay. The levels of CORT, ACTH and CRF were measured with an enzyme-linked immunosorbent assay (ELISA) kit according to the manufacturer’s instructions (Cayman Chemical, Ann Arbor, MI, USA).

### Dexamethasone (Dex) Suppression Test

The mice were injected (i.p.) with Dex (Sigma) at a concentration of 0.1 mg/kg to achieve partial (approximately 50%) suppression of the HPA axis. Injections were performed between 09:00 h and 10:00 h. Six hours later they were rapidly decapitated for blood collection to examine the level of CORT (Vicentini et al., 2009).

### Responses of HPA Axis to Acute Mild Restraint Stress (AMRS)

The animals were restrained within Plexiglas restraint tubes for 15 min to induce AMRS (Livingstone et al., 2014). They were returned to their home cages and a further blood samples were obtained at 30, 60 or 90 min following the start of the AMRS for examination of plasma CORT and ACTH. Blood samples were collected at only one time point following the end of the AMRS to avoid confounding effects of repeated stress. The blood was obtained rapidly (within 30 s) by tail-nick.

### Histological Examination

Mice were anesthetized with chloral hydrate (400 mg/kg, i.p.) and then perfused with 4% paraformaldehyde. Brains were removed and continuously fixed in 4% paraformaldehyde for 24 h, and then were transferred into 30% sucrose. After the brains completely sunk to the bottom in 30% sucrose, the hypothalamic PVN area located at 0.58 to −1.08 mm from bregma (Ghosal et al., 2017) was coronally sectioned on a freezing microtome (Leica CM3050S; Leica Microsystems, Germany). The coronal sections were pre-incubated 60 min with PBS containing 0.3% Triton X-100 and 3% normal horse serum and then incubated in the antibodies of mouse anti-σ_1_R (1:200; Santa Cruz, CA, USA) at 4°C overnight. After PBS rinses, the sections were treated with a biotin-labeled horse anti-mouse IgG antibody (1:200, Vector Laboratories) for 2 h. Immunoreactivities were visualized by avidin-biotin horseradish peroxidase complex and observed using a light microscope with a CCD camera (Olympus DP70).

### Western Blotting Analysis

Protein was extracted from PVN. The amount of protein was determined using a bicinchoninic acid (BCA) protein assay kit (Pierce, IL, USA). Protein (40 μg) was separated by 8% acrylamide denaturing gels (SDS-PAGE) and transferred to membranes. The membranes were incubated with the antibodies of rabbit monoclonal anti-GR (1:1000, Abcam, Cambridge, UK), anti-GR phosphorylation (1:1000, Abcam), anti-PKC phosphorylation (1:1000, Abcam), anti-CREB phosphorylation (1:500, Abcam), anti-PKA (1:1000, Cell Signaling Technology, Inc., Boston, MA, USA) or anti-PKA phosphorylation (1:1000, Abcam). After TBST buffer rinses, the membranes were incubated with goat anti-rabbit antibodies (1:5000, Millipore, Billerica, MA, USA) and developed using an enhanced chemiluminescence detection kit (Pierce, IL, USA). Following visualization, the blots were stripped by incubation in stripping buffer (Pierce, IL, USA) for 15 min, and then incubated with the antibodies of anti-PKC, anti-CREB or anti-PKA. The western blot bands were scanned and analyzed with the Image J analysis software package (NIH). Densitometric value of phosphorylated protein normalized by total protein was normalized again by control level.

### Reverse Transcription-Polymerase Chain Reaction (RT-PCR)

Total RNA was isolated from the PVN with TRIzol reagent (Invitrogen, Camarillo, CA, USA) and reverse-transcribed into cDNA using a Prime Script RT reagent kit (Takara, China) for quantitative PCR (ABI Step One Plus, Foster City, CA) in the presence of a fluorescent dye (SYBR Green I, Takara, China). The relative expression of genes was determined using the 2^−∆∆ct^ method with normalization to GAPDH and β-actin in each sample. The results were averaged from four sets of independent experiments. The primer sets used for *GR* (forward 5′-AGCTCCCCCTGGTAGAGAC-3′; reverse 5′-GGTGAAGACGCAGAAACCTT-3′), *Nr3c1* (forward 5′-ACCTGGAAGCTCGAAAAACGA-3′; reverse 5′-CAGCAGTGACACCAGGGTAG-3′) and *CRF* (forward 5′-AAGAAATTCAAGGGCTGCGG-3′; reverse 5′-GGAGAAGAGAGCGCCCCTAA-3′) were designed according to the publication (Ayuob et al., 2016; Karisetty et al., 2017).

### Statistical Analysis

Data were retrieved and processed with the software PulseFit (HEKA Elektronik). The group data were expressed as the means ± standard error (SEM). All statistical analyses were performed using SPSS software, version 16.0 (SPSS Inc., USA). Differences among means were analyzed using either a one-way or two-way analysis of variance (ANOVA), followed by the Bonferroni *post hoc* analysis for multiple comparisons, where appropriate. Differences at *P* < 0.05 were considered statistically significant.

## Results

### Effects of σ_1_R Deficiency on the Activity of HPA Axis

**Figure 1:**
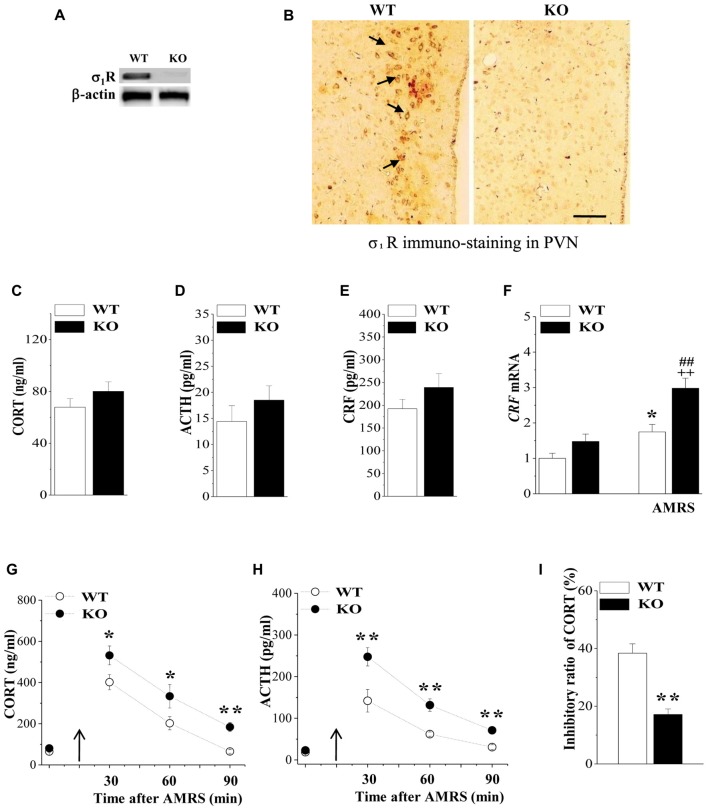
Expression of Sigma-1 receptor (σ_1_R) in paraventricular nuclei (PVN) and the activity of the hypothalamic-pituitary-adrenocortical (HPA) axis. **(A)** Representative blots of σ_1_R protein in the PVN of wild-type (WT) mice (WT) and σ_1_R knockout (σ_1_R-KO) mice (KO). **(B)** Representative photomicrograph of σ_1_R immunohistochemical staining. σ_1_R-positive principal neurons (arrows) in the PVN of WT mice. Scale bars = 25 μm. **(C–E)** Activities of the HPA axis in WT mice and σ_1_R-KO mice. Bar graphs show the basal levels of serum corticosterone (CORT), adrenocorticotropic hormone (ACTH) and corticotrophin releasing factor (CRF). **(F)** The levels of *CRF* mRNA under basal conditions or at 30 min after acute mild restraint stress (AMRS). *P < 0.05 vs. WT mice; ^##^*P* < 0.01 vs. σ_1_R-KO mice; ^++^*P* < 0.01 WT mice-subjected to AMRS (two-way analysis of variance (ANOVA)). **(G,H)** Each point represents the level of serum CORT or ACTH after AMRS. A solid arrow indicates the time of the AMRS exposure. *P < 0.05 and **P < 0.01 vs. WT mice-subjected to AMRS (repeated-measures ANOVA). **(I)** Bar graphs show the reduced level of CORT and ACTH at 6 h after the injection of dexamethasone (Dex; 0.1 mg/kg) compared to those at 48 h before the injection (basal levels). The inhibitory ratio was calculated with the following formula: (levels after Dex injection/basal levels) × 100. **P < 0.01 vs. WT mice (*t* test).

The Western blotting analysis showed the expression of σ_1_R protein at approximately 28 kD in the hypothalamic PVN of WT mice and a lack of σ_1_R protein in σ_1_R KO (σ_1_R-KO) mice (Figure 1A). Immunohistochemical observation further confirmed the σ_1_R expression in the PVN neurons of WT mice, but not σ_1_R-KO mice (Figure 1B). Consistent with earlier reports (Alonso et al., 2000), the σ_1_R protein was mainly located in neuronal perikarya.

HPA axis dysregulation is well known to be the most prominent endocrine mechanism in affective disorders. We initially examined the influence σ_1_R deficiency on the basal activity of HPA axis by analyzing the levels of serum CORT, ACTH and CRF as well as the expression of CRF in PVN. As same as reported previously by Sha et al. (2015), the level of serum CORT did not differ between σ_1_R-KO mice and WT mice (*P* > 0.05, *n* = 8; Figure 1C). Although the levels of serum ACTH (*P* > 0.05, *n* = 8; Figure 1D) and CRF (*P* > 0.05, *n* = 8; Figure 1E), or the level of *CRF* mRNA (*P* > 0.05, *n* = 8; Figure 1F) tended to be higher in σ_1_R-KO mice, these differences were not significant compared to the WT group.

CRF synthesis and release from the hypothalamic PVN is the prime mediator of the HPA axis response to stress, which acts synergistically to stimulate the secretion of ACTH, leading to an increase in circulating glucocorticoids. Subsequently, we investigated the response of the HPA axis to 15 min AMRS in σ_1_R-KO mice and WT mice. Notably, the levels of CORT (Figure 1G), ACTH (Figure 1H) and *CRF* mRNA (Figure 1F) were affected by AMRS (CORT: *F*_(1,60)_ = 23.24, *P* < 0.001; ACTH: *F*_(2,44)_ = 23.32, *P* < 0.001; *CRF* mRNA: *F*_(1,28)_ = 26.61, *P* < 0.001) or σ_1_R deficiency × AMRS (CORT: *F*_(1,60)_ = 9.35, *P* = 0.003; ACTH: *F*_(1,60)_ = 9.09, *P* = 0.004; *CRF* mRNA: *F*_(1,28)_ = 34.60, *P* < 0.001). After the exposure to AMRS, the levels of serum CORT (at 30/60 min: *P* < 0.05, *n* = 8; at 90 min: *P* < 0.01, *n* = 8) and ACTH (at 30/60/90 min: *P* < 0.01, *n* = 8) in σ_1_R-KO mice were higher than those in WT mice. In addition, the level of *CRF* mRNA at 30 min after the exposure to AMRS was increased in WT mice compared to the basal level (*P* < 0.01, *n* = 8). In σ_1_R-KO mice, the AMRS-induced increase in *CRF* mRNA was approximately 2-fold higher than that in WT mice (*P* < 0.01, *n* = 8). These results indicate that σ_1_R deficiency facilitates the stress response of the HPA axis, which results in the long-lasting hyperactivity of HPA axis after the exposure to AMRS.

Circulating CORT is critical for recovery from stress conditions, because it induces a negative feedback regulation of the HPA axis; for example, it decreases the production of CRF, the secretion of CRF and ACTH. The subsequent experiments used the Dex suppression test to examine the influence of σ_1_R deficiency on the GR-mediated negative feedback regulation in the activity of HPA axis. At 6 h after the injection of Dex (0.1 mg/kg), the level of serum CORT in WT mice were decreased by approximately 40%, compared to the basal level at 48 h before the Dex injection (Figure 1I). In contrast, the percentage of Dex-reduced CORT in σ_1_R-KO mice was approximately one third the value measured in WT mice (*P* < 0.01, *n* = 8). These results indicate that σ_1_R deficiency attenuates the GR-mediated feedback inhibition of the HPA axis, which leads to the long-lasting hyperactivity of HPA axis after the exposure to AMRS.

### Effects of σ_1_R Deficiency on GR Expression and Activity in PVN

**Figure 2:**
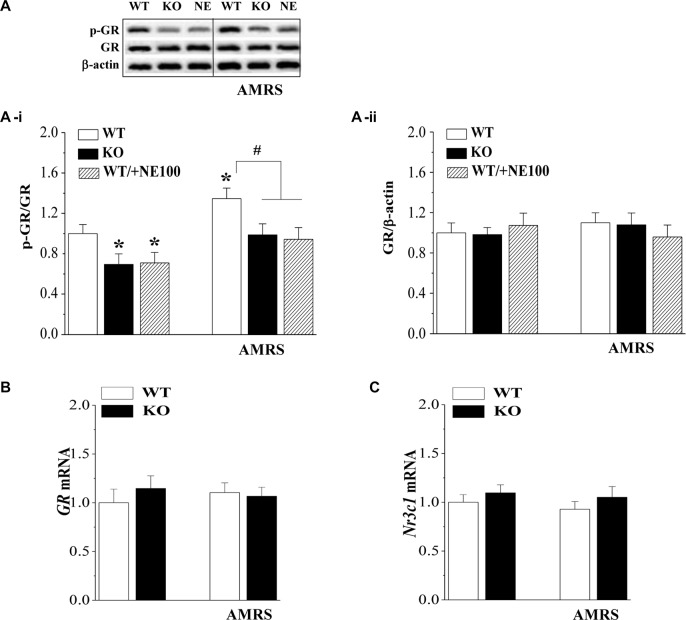
Effects of σ_1_R deficiency on glucocorticoid receptor (GR) expression and activity in PVN. **(A)** Levels of phospho-GR in PVN of WT mice (WT), σ_1_R-KO mice (KO) and NE100-treated WT mice (NE) under basal conditions and after AMRS. The densitometric value of phospho-GR was normalized to that of GR protein **(A-i)**, and the value GR protein was normalized that of β-actin **(A-ii)**, and these values were again normalized to the control levels obtained from WT mice. *P < 0.05 vs. WT mice; ^#^*P* < 0.05 vs. WT mice-subjected to AMRS (two-way ANOVA). **(B,C)** Levels of *GR* mRNA and *Nr3c1* mRNA in the PVN of WT mice and σ_1_R-KO mice.

The GR is responsible for the negative feedback effects of Dex and is a main effector in the restoration of stress homeostasis (Barden, 2004). To investigate the mechanisms underlying the deficient GR-mediated inhibition of the HPA axis in σ_1_R-KO mice, we examined the GR expression and phosphorylation (phospho-GR) in PVN under basal condition and after the exposure to AMRS (Figure 2A). As shown in Figure 2A-i, the levels of phospho-GR were affected by σ_1_R deficiency (*F*_(1,44)_ = 15.00, *P* < 0.001) and AMRS (*F*_(1,44)_ = 11.76, *P* < 0.001), but not σ_1_R deficiency × AMRS (*F*_(1,44)_ = 0.19, *P* = 0.67). In comparison with WT mice, the basal level of phospho-GR in σ_1_R-KO mice (*P* < 0.05, *n* = 8) and WT mice treated with the injection (i.c.v.) of the σ_1_R antagonist NE100 for 3 days (*P* < 0.05, *n* = 8) was reduced by approximately 20%–30% without the change in the level of GR protein (*P* > 0.05, *n* = 8; Figure 2A-ii). At 30 min after the exposure to AMRS, the level of phospho-GR was elevated by approximately 30%–40% in WT mice (*P* < 0.05, *n* = 8). In σ_1_R-KO mice or NE100-treated WT mice exposed to AMRS the level of phospho-GR was slightly elevated, but these increases when compared with the basal levels failed to reach the significance (*P* > 0.05, *n* = 8). Thus, the level of phospho-GR after the exposure to AMRS in σ_1_R-KO mice or NE100-treated WT mice was lower than that in WT mice (*P* < 0.05, *n* = 8). In addition, the levels of *GR* mRNA (*P* > 0.05, *n* = 8; Figure 2B) and *Nr3c1* mRNA (*P* > 0.05, *n* = 8; Figure 2C) under basal condition or after the exposure to AMRS did not significantly differ between WT mice and σ_1_R-KO mice. These results indicate that σ_1_R deficiency suppresses the phosphorylation of GR probably leading to dysfunction of GR.

### Involvement of PKC Signaling in the Hyperactivity of HPA Axis in σ_1_R-KO Mice

**Figure 3:**
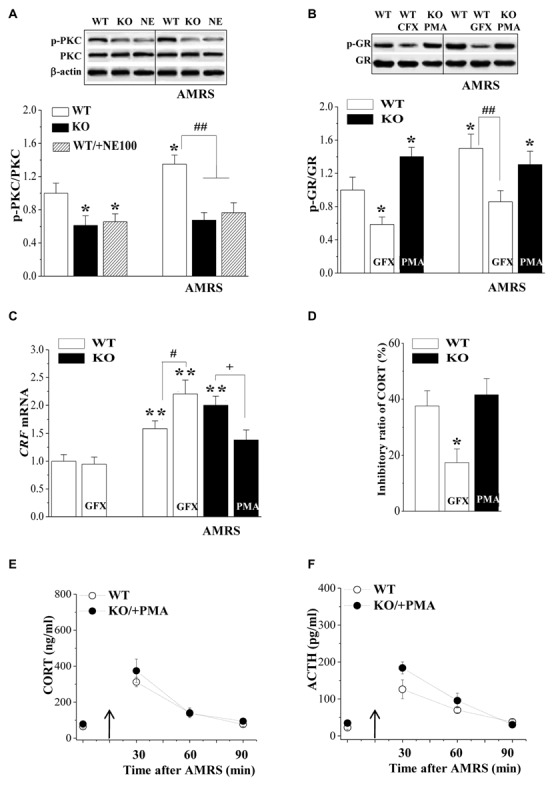
Involvement of protein kinase C (PKC) signaling in the hyperactivity of HPA axis in σ_1_R-KO mice. **(A)** Levels of phospho-PKC in the PVN of WT mice (WT), σ_1_R-KO mice (KO) and NE100-treated WT mice under basal conditions and after AMRS. *P < 0.05 vs. WT mice; ^##^*P* < 0.01 vs. WT mice-subjected to AMRS (two-way ANOVA). **(B,C)** Levels of phospho-GR and *CRF* mRNA under basal conditions and after AMRS. *P < 0.05 and **P < 0.01 vs. WT mice; ^#^*P* < 0.05 and ^##^*P* < 0.01 vs. WT mice-subjected to AMRS; ^+^*P* < 0.05 vs. σ_1_R-KO mice-subjected to AMRS (two-way ANOVA). **(D)** Levels of CORT at 6 h after Dex injection in WT mice treated with GF109203X (GFX) or σ_1_R-KO mice treated with PMA. *P < 0.05 vs. WT mice (repeated-measures ANOVA). **(E,F)** Level of serum CORT or ACTH after AMRS.

To further explore the molecular mechanisms of the reduced phospho-GR in σ_1_R-KO mice, we examined the level of phosphorylated PKC (phospho-PKC) in the PVN, which has been associated with the regulation of phospho-GR (Kotitschke et al., 2009). Similarly, the levels of phospho-PKC were affected by σ_1_R deficiency (*F*_(1,44)_ = 28.13, *P* < 0.001; Figure 3A) or AMRS (*F*_(1,44)_ = 5.45, *P* = 0.02) rather than σ_1_R deficiency × AMRS (*F*_(1,44)_ = 1.99, *P* = 0.17). The levels of phospho-PKC in σ_1_R-KO mice and NE100-treated WT mice were reduced in comparison with WT mice (*P* < 0.05; *n* = 8). Furthermore, the exposure to AMRS could elevate the level of phospho-PKC in WT mice (*P* < 0.05, *n* = 8), but not in σ_1_R-KO mice or NE100-treated WT mice (*P* > 0.05, *n* = 8).

Importantly, the injection (i.c.v.) of the PKC activator PMA for 3 days in σ_1_R-KO mice caused a higher level of phospho-GR than that in WT mice (*P* < 0.05, *n* = 8; Figure 3B) and recovered the AMRS-induced increase of phospho-GR (vs. WT mice, *P* > 0.05, *n* = 8). In contrast, the injection (i.c.v.) of the PKC inhibitor GF109203X in WT mice decreased the basal level of phospho-GR (*P* < 0.05, *n* = 8) and the AMRS-induced increase of phospho-GR (*P* < 0.01, *n* = 8).

Although the injection (i.c.v.) of GF109203X failed to affect the basal level of *CRF* mRNA in WT mice (*P* > 0.05, *n* = 8; Figure 3C), it enhanced the AMRS-induced increase in the level of *CRF* mRNA (*P* < 0.05, *n* = 8). The treatment of σ_1_R-KO mice with PMA attenuated the AMRS-induced increase in the level of *CRF* mRNA compared to vehicle-treated σ_1_R-KO mice (*P* < 0.05, *n* = 8). Additionally, treating σ_1_R-KO mice with PMA recovered the percentage of Dex-reduced CORT (*vs*. WT mice *P* > 0.05, *n* = 8; Figure 3D). As expected, the inhibition of PKC by GF109203X in WT mice attenuated the percentage of Dex-reduced CORT (*P* < 0.05, *n* = 8). In comparison with WT mice, the activation of PKC by PMA in σ_1_R-KO mice corrected the enhancement of AMRS-induced increases in CORT (*F*_(1,14)_ = 0.77, *P* = 0.39; Figure 3E) and ACTH (*F*_(1,14)_ = 3.76, *P* = 0.07; Figure 3F). These results indicate that σ_1_R deficiency suppresses GR phosphorylation by reducing PKC activity, which suppresses the GR-mediated feedback inhibition of the HPA axis and facilitates the responsiveness of the HPA axis to the AMRS.

### Involvement of PKA-CREB Signaling in the Hyperactivity of HPA Axis in σ_1_R-KO Mice

**Figure 4:**
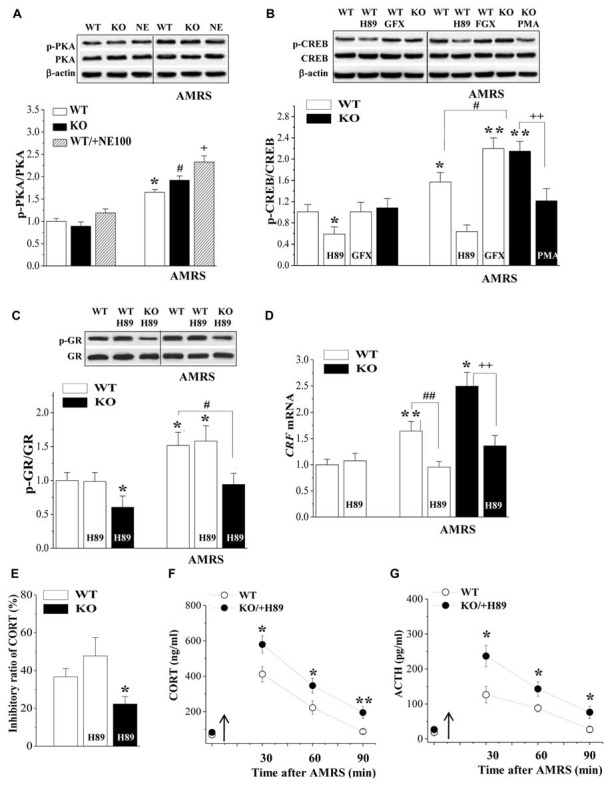
Involvement of PKA-cAMP-response element binding protein (CREB) signaling in the hyperactivity of the HPA axis in σ_1_R-KO mice. **(A)** Levels of phospho-PKA under basal conditions and after AMRS. *P < 0.05 vs. WT mice; ^#^*P* < 0.05 vs. σ_1_R-KO mice; ^+^*P* < 0.05 vs. NE100-treated WT mice (two-way ANOVA). **(B)** Levels of phospho-CREB under basal conditions and after AMRS. *P < 0.05 and **P < 0.01 vs. WT mice; ^#^*P* < 0.05 vs. WT mice-subjected to AMRS; ^++^*P* < 0.01 vs. σ_1_R-KO mice-subjected to AMRS (two-way ANOVA). **(C,D)** Levels of phospho-GR and *CRF* mRNA under basal conditions and after AMRS. *P < 0.05 and **P < 0.01 vs. WT mice; ^#^*P* < 0.05 and ^##^*P* < 0.01 vs. WT mice-subjected to AMRS; ^++^*P* < 0.01 vs. σ_1_R-KO mice-subjected to AMRS (two-way ANOVA). **(E)** Levels of serum CORT at 6 h after Dex injection. *P < 0.05 vs. WT mice (one-way ANOVA). **(F,G)** Levels of serum CORT or ACTH after AMRS. *P < 0.05 and **P < 0.01 vs. WT mice (repeated-measures ANOVA).

The PKA-CREB pathway is known to enhance CRF gene transcription in hypothalamic cells (Kageyama et al., 2010). Subsequently, we examined the levels of phosphorylated PKA (phospho-PKA) and CREB (phospho-CREB) in the PVN. There was a main effect of AMRS on the levels of phospho-PKA (*F*_(1,44)_ = 11.13, *P* = 0.002; Figure 4A) and phospho-CREB (*F*_(1,28)_ = 22.88, *P* < 0.001; Figure 4B). However, the levels of phospho-PKA and phospho-CREB failed to be altered by the σ_1_R deficiency (p-PKA: *F*_(1,44)_ = 0.98, *P* = 0.33; p-CREB: *F*_(1,28)_ = 3.63, *P* = 0.07) or σ_1_R deficiency × AMRS (p-PKA: *F*_(1,44)_ = 0.70, *P* = 0.41; p-CREB: *F*_(1,28)_ = 2.24, *P* = 0.15). After the exposure to AMRS, the levels of phospho-PKA were significantly elevated in either WT mice (*P* < 0.05, *n* = 8) or σ_1_R-KO mice (*P* < 0.05, *n* = 8) and NE100-treated WT mice (*P* < 0.05, *n* = 8). The basal level of phospho-PKA or the level of AMRS-increased phospho-PKA did not significantly differ between WT mice and σ_1_R-KO mice or NE100-treated WT mice (*P* > 0.05, *n* = 8). The basal level of phospho-CREB in σ_1_R-KO mice had no significant difference from WT mice (*P* > 0.05, *n* = 8), whereas the exposure to AMRS induced a stronger phospho-CREB in σ_1_R-KO mice than in WT mice (*P* < 0.01, *n* = 8). In WT mice, the injection (i.c.v.) of the PKA inhibitor H89 for 3 days reduced the basal level of phospho-CREB (*P* < 0.05, *n* = 8) and the AMRS-induced increase in level of phospho-CREB (*P* < 0.01, *n* = 8). Although the injection (i.c.v.) of GF109203X did not affect the basal phospho-CREB level in WT mice (*P* > 0.05, *n* = 8), it enhanced the AMRS-induced increase in level of phospho-CREB (*P* < 0.01, *n* = 8). Moreover, the PMA-injection (i.c.v.) in σ_1_R-KO mice corrected the enhancement of AMRS-increased phospho-CREB (*P* < 0.01, *n* = 8). Thus, the findings indicate that σ_1_R deficiency via the down-regulation of PKC promotes the AMRS-induced activation of CREB.

The injection (i.c.v.) of H89 in WT mice failed to alter the basal level of phospho-GR (*P* > 0.05, *n* = 8; Figure 4C) and AMRS-increased phospho-GR (*P* > 0.05, *n* = 8). The basal level of phospho-GR (*P* < 0.05, *n* = 8) and the level of AMRS-increased phospho-GR (*P* < 0.01, *n* = 8) in H89-treated σ_1_R-KO mice were still lower than those in WT mice. Although the H89-injection (i.c.v.) in WT-mice had no effect on the basal level of *CRF* mRNA (*P* > 0.05, *n* = 8; Figure 4D), it significantly inhibited the AMRS-increased *CRF* mRNA (*P* < 0.01, *n* = 8). Similarly, the H89-injection (i.c.v.) in σ_1_R-KO mice could prevent the AMRS-induced increase in the *CRF* mRNA (*P* < 0.01, *n* = 8). The H89-injection (i.c.v.) did not alter the percentage of Dex-reduced CORT in WT mice (*P* > 0.05, *n* = 8; Figure 4E), thus the percentage of Dex-reduced CORT in H89-treated σ_1_R-KO mice was reduced compared to WT mice (*P* < 0.05, *n* = 8). Furthermore, the AMRS-induced increases in the levels of CORT (*F*_(1,14)_ = 21.29, *P* < 0.001; Figure 4F) and ACTH (*F*_(1,14)_ = 22.98, *P* < 0.001; Figure 4G) in H89-treated σ_1_R-KO mice were higher than those in WT mice. These results indicate that the CREB signaling is involved in the enhancement of AMRS-induced CRF expression in σ_1_R-KO mice.

### Relation of HPA Axis Hyperactivity to Depressive-Like Behaviors in σ_1_R-KO Mice

**Figure 5:**
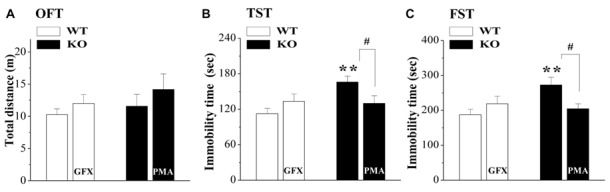
Relationship between HPA axis hyperactivity and depressive-like behaviors in σ_1_R-KO mice. **(A–C)** Bar graphs show the distance traveled in the open-field test (OFT) **(A)** the immobility time during the tail suspension test (TST) **(B)** or forced swim test (FST) **(C)** in WT mice treated with GF109203X (GFX) or σ_1_R-KO mice treated with PMA. **P < 0.01 vs. WT mice; ^#^*P* < 0.05 vs. σ_1_R-KO mice (one-way ANOVA).

Spontaneous motor and depression-like behaviors were examined in an OFT, a FST and a TST (*n* = 8; per experimental group). As shown in Figure 5A, the distance traveled in the OFT did not significantly differ between WT mice and σ_1_R-KO mice (*P* > 0.05). Compared with WT mice, the immobility times in the TST (*P* < 0.01; Figure 5B) and FST (*P* < 0.01; Figure 5C) were increased in σ_1_R-KO mice. To explore the causal relationship between the hyperactivity of the HPA axis and the depressive-like behaviors, σ_1_R-KO mice were given the injection (i.c.v.) of PMA or WT mice were treated with the injection (i.c.v.) of GF109203X for three consecutive days. The results showed the activation of PKC in σ_1_R-KO mice could correct these increases in the immobility times of TST (*P* < 0.05) and FST (*P* < 0.05) without changing the spontaneous motor response (*P* > 0.05). Although the injection (i.c.v.) of GF109203X tended to increase the immobility time in the TST (*P* > 0.05) and FST in WT mice, these differences were not significant compared to the vehicle-treated WT controls (*P* > 0.05).

## Discussion

The present study provides, for the first time, *in vivo* evidence to show that σ_1_R deficiency in CRF cells attenuates the GR-mediated feedback inhibition of the HPA axis and facilitates the stress response of the HPA axis by down-regulation of PKC signaling to suppress the GR phosphorylation, which results in depressive-like behaviors.

### GR Activation Is Suppressed by Down-Regulation of PKC in σ_1_R-KO Mice

Several lines of evidence suggest that the GR can cross-talk with steroid receptors, which may occur in specialized membrane lipid rafts or caveolae microdomains, which results in the site-specific phosphorylation and transactivation of an endogenous gene (Kotitschke et al., 2009). The σ_1_R, which is a neurosteroid receptor, is highly clustered in lipid rafts or caveolae microdomains (Hayashi and Su, 2005). The translocation of σ_1_R from lipid droplets on the endoplasmic reticulum to the plasma membrane when stimulated by agonists induces the activation of PKC (Morin-Surun et al., 1999). One interesting observation in this study is that the genomic σ_1_R deficiency or the pharmacological blockade of σ_1_R all decreased the PKC phosphorylation in PVN with the decline of GR phosphorylation. The inhibition of PKC was able to suppress the GR phosphorylation without changing the GR protein level. Notably, the level of GR phosphorylation in σ_1_R-KO mice treated with a PKC activator was higher than that in WT mice. The binding of glucocorticoids or Dex can phosphorylate the GR (Adzic et al., 2009; Brossaud et al., 2017). Interestingly, the exposure to AMRS could enhance the phosphorylation of GR and PKC in WT mice, but not in σ_1_R-KO mice and WT mice treated with σ_1_R antagonist or PKC inhibitor. However, the exposure to AMRS could not further elevate the level of GR phosphorylation in σ_1_R-KO mice treated with PKC activator. The earlier studies reported the interaction between GR and PKC signaling pathway (Pérez-Martínez et al., 1998; Cote-Vélez et al., 2005). The PKC signaling modulates positively the GR activity (Cote-Vélez et al., 2008). Thus, it is conceivable that that the down-regulation of PKC in σ_1_R-KO mice suppresses the GR phosphorylation leading to the decline of GR activity.

### GR Dysfunction Leads to the Hyperactivity of HPA Axis in σ_1_R-KO Mice

**Figure 6:**
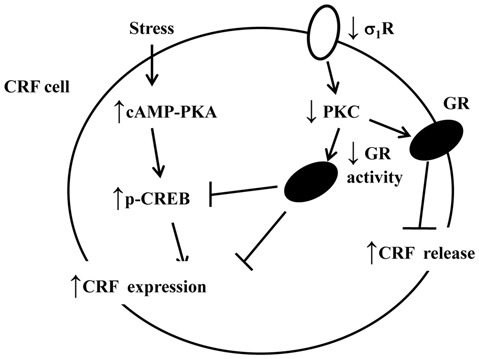
The hypothesized molecular mechanisms underlying the reduced GR-mediated feedback inhibition of the HPA axis and hyperactivity of the HPA axis after exposure to an AMRS in σ_1_R-KO mice. ↑: increase; ↓: decrease.

The corticosteroid effects on CRF neurons have been attributed to membrane-associated GR-mediated nongenomic steroid action and “classical” intracellular GR-mediated transcriptional steroid action (Tasker and Herman, 2011). The activity of HPA axis is subject to the negative feedback control of circulating glucocorticoids, which involves the suppression of rapid CRF release and delayed CRF production in these neurons, respectively (Kageyama and Suda, 2009). The activation of membrane GR results in the generation of a retrograde signal that traverses back across the synaptic cleft to the axon terminals of excitatory suppresses the release of glutamate onto CRF neurons, suppressing the release of CRF. A principal finding in this study is that the percentage of Dex-reduced CORT in σ_1_R-KO mice was lower than that in WT mice, which was corrected by the PKC activator. Moreover, the treatment of WT mice with PKC inhibitor attenuated the percentage of Dex-reduced CORT. The KO of GR in PVN resulted in an increase in the levels of ACTH and CORT during the circadian peak and in response to restraint (Schmidt et al., 2009; Laryea et al., 2013). Thus, it is highly likely that the decline of GR activity in σ_1_R-KO mice is able to reduce the Dex-induced feedback inhibition of HPA axis (Figure 6). The results in the present study give an indication that the decrease in Dex-induced feedback inhibition of HPA axis in patients with depression is caused, at least partly, by the down-regulation of GR.

CRF primary transcript is increased after 30 min of restraint, whereas the increased CRF transcript returned to basal levels by 90 min, despite persistent stressor (Shepard et al., 2005). Another important finding in this study is that the exposure of σ_1_R-KO mice to AMRS caused a persistent (over 90 min) hyperactivity of the HPA axis with an increase in *CRF* mRNA, although the basal activity of the HPA axis and level of CRF expression did not significantly differ between σ_1_R-KO mice and WT mice. The enhancement of AMRS-increased CRF expression in σ_1_R-KO mice was sensitive to the PKC activator. Moreover, either the blockade of σ_1_R or the inhibition of PKC in WT mice could enhance the AMRS-increased CRF expression. The repression of CRF gene expression by glucocorticoids is reportedly mediated by the inhibition of CRF gene transcription (Morin-Surun et al., 1999). There are a number of glucocorticoid regulatory regions in the sequence of CRF promoter where GRs are able to bind. Malkoski and Dorin (1999) demonstrated a specific inhibitory region of the CRF promoter activity by glucocorticoids. In mouse fibroblast cells transfected with CORT-induced chloramphenicol acetyltransferase (CAT) plasmid, the σ_1_R agonists decrease CORT-induced gene transcription in a concentration- and time-dependent manner (Skuza et al., 2011). The activation of σ_1_R decreases the binding of the GR complex to DNA, and its inhibitory effect depends partly on the PLC/PKC pathway (Budziszewska, 2002). Thus, it is indicated that the decline of GR activity in σ_1_R-KO mice is able to enhance the AMRS-increased CRF biosynthesis (Figure 6). Further experiments will be required to determine whether the σ_1_R deficiency affects the binding of GR to the sequence of CRF promoter.

The activation of PKA signaling can stimulate the CRF gene promoter activity via an identified CREB activation. After the exposure to AMRS, the level of phospho-PKA in σ_1_R-KO mice did not significantly differ from WT mice, whereas the level of phospho-CREB in σ_1_R-KO mice was higher than in WT mice, and this increase was corrected by the PKC activator. However, the inhibition of PKC has been reported to reduce the CREB phosphorylation (Ishigame et al., 2016). Treatment with a PKC inhibitor can reduce the morphine withdrawal-triggered increase in CREB activation (Martín et al., 2011). In this study, treating WT mice with the PKC inhibitor did not alter the basal level of phospho-CREB, but it significantly enhanced the AMRS-increased phospho-CREB. On the other hand, glucocorticoids can suppress CREB phosphorylation and CRF biosynthesis (Légrádi et al., 1997), and the inhibition of PKC attenautes glucocorticoid-induced gene transcription (Budziszewska et al., 2000). Therefore, one possible explanation is that the GR-mediated inhibition of the CREB phosphorylation in σ_1_R-KO mice is reduced, which may enhance AMRS-increased CRF biosynthesis (Figure 6). However, it is not known how the GR dysfunction in σ_1_R-KO mice facilitates the AMRS-induced CREB phosphorylation.

### Association of the Hyperactivity of HPA Axis with Depressive-Like Behaviors in σ_1_R-KO Mice

The σ_1_R agonists have been demonstrated to decrease the immobility time in the TST and the FST (Ukai et al., 1998; Urani et al., 2001; Skuza and Rogóz, 2002). Depression and anxiety occur in over 50% of patients with Cushing’s syndrome (Dimopoulou et al., 2013) and up to 20% of patients who receive exogenous glucocorticoids for immunosuppressive therapy (Kenna et al., 2011). Chronic glucocorticoid administration also promotes depression-like and anxiety-like behaviors in animals (Sterner and Kalynchuk, 2010). The “antidepressant-like” activity of σ_1_R ligands can be directly connected with GR function (Skuza et al., 2008). Our results supported the idea, because the injection (i.c.v.) of a PKC activator in σ_1_R-KO mice could correct the increase in the immobility time in the TST or FST. Glucocorticoid can induce oxidative load in the brain with significant increases in pro-oxidant (lipid peroxidation and nitrite levels) markers and a substantial decline in the anti-oxidant defense (catalase and reduced glutathione levels) system, indicating that stress hormones directly induce the brain oxidative damage (Gupta et al., 2015). Mori et al. (2012) reported that the σ_1_R deficiency elevates the ROS level. Antidepressant drugs are known to inhibit the hyperactivity of HPA axis, which is often observed in depression. Thus, the hyperactivity of HPA axis in σ_1_R-KO mice is responsible for the onset of their depressive-like behaviors. However, the inhibition of PKC alone in WT mice failed to increase significantly the immobility time in TST or FST. Furthermore, the basal activity of HPA axis and level of CRF expression in σ_1_R-KO mice had no significant difference from WT mice, although σ_1_R-KO mice showed an increase in the immobility time in the TST or FST. Recently, some studies have reported that bipolar disorder is associated with small increases of cortisol levels (Girshkin et al., 2014) or dysfunction of HPA axis activity (Belvederi Murri et al., 2016), suggesting that the various dysregulation of HPA axis activity might partly mediate the mood disturbances. On the other hand, the antidepressant effects of the σ_1_R agonists in several behavioral models have been associated with their enhancement of glutamatergic neuronal functions (Cobos et al., 2008). Selective σ_1_R ligands can potentiate the neuronal response to NMDA (Martina et al., 2007) and the Ca^2+^ influx across the NMDA receptor (NMDAr; Cai et al., 2008), or enhances NMDAr trafficking to the plasma membrane (Pabba et al., 2014). Indeed, the density of NMDA-induced current is reduced in the hippocampal neurons of σ_1_R-KO mice (Sha et al., 2013). Sha et al. (2015) reported an antidepressant effect of the NMDAr agonist in σ_1_R-KO mice. However, some selective NMDAr antagonists that also bind to sigma receptors (Hashimoto and London, 1993) have been associated with antidepressant effects in animal models and human of depression (Serafini et al., 2014). Thus, further studies are needed to provide greater insights into the more complex mechanism underlying the production of depressive-like behaviors by σ_1_R deficiency.

In summary, the σ_1_R deficiency in CRF neurons reduces the GR-mediated feedback inhibition of the HPA axis and facilitates the response of the HPA axis to stress via the down-regulation of PKC, which leads to the long-lasting hyperactivity of HPA axis and the production of depressive-like behaviors. Results in the present study can help for understanding the molecular mechanisms underlying the production of depressive-like phenotype in σ_1_R^−/−^ mice and the antidepressant effects of the σ_1_R agonists.

## Author Contributions

TD and SZ designed and performed the experiment. JH and TZ analyzed and interpreted the data and wrote the manuscript. LC designed and supervised the studies and critically revised the manuscript. All authors approved the final version for publication.

## Conflict of Interest Statement

The authors declare that the research was conducted in the absence of any commercial or financial relationships that could be construed as a potential conflict of interest.
